# Variation in the PaO_2_/FiO_2 _ratio with FiO_2_: mathematical and experimental description, and clinical relevance

**DOI:** 10.1186/cc6174

**Published:** 2007-11-07

**Authors:** Dan S Karbing, Søren Kjærgaard, Bram W Smith, Kurt Espersen, Charlotte Allerød, Steen Andreassen, Stephen E Rees

**Affiliations:** 1 Center for Model-based Medical Decision Support, Department of Health Science and Technology, Aalborg University, Fredrik Bajers Vej 7, E4-215, DK-9220 Aalborg East, Denmark; 2 Anaesthesia and Intensive Care, Region North Jutland, Aalborg Hospital, Aarhus University, DK-9000 Aalborg, Denmark; 3 Department of Intensive Care, Rigshospitalet, University of Copenhagen, DK-2100 Copenhagen East, Denmark

## Abstract

**Introduction:**

Previous studies have shown through theoretical analyses that the ratio of the partial pressure of oxygen in arterial blood (PaO_2_) to the inspired oxygen fraction (FiO_2_) varies with the FiO_2 _level. The aim of the present study was to evaluate the relevance of this variation both theoretically and experimentally using mathematical model simulations, comparing these ratio simulations with PaO_2_/FiO_2 _ratios measured in a range of different patients.

**Methods:**

The study was designed as a retrospective study using data from 36 mechanically ventilated patients and 57 spontaneously breathing patients studied on one or more occasions. Patients were classified into four disease groups (normal, mild hypoxemia, acute lung injury and acute respiratory distress syndrome) according to their PaO_2_/FiO_2 _ratio. On each occasion the patients were studied using four to eight different FiO_2 _values, achieving arterial oxygen saturations in the range 85–100%. At each FiO_2 _level, measurements were taken of ventilation, of arterial acid–base and of oxygenation status. Two mathematical models were fitted to the data: a one-parameter 'effective shunt' model, and a two-parameter shunt and ventilation/perfusion model. These models and patient data were used to investigate the variation in the PaO_2_/FiO_2 _ratio with FiO_2_, and to quantify how many patients changed disease classification due to variation in the PaO_2_/FiO_2 _ratio. An *F *test was used to assess the statistical difference between the two models' fit to the data. A confusion matrix was used to quantify the number of patients changing disease classification.

**Results:**

The two-parameter model gave a statistically better fit to patient data (*P *< 0.005). When using this model to simulate variation in the PaO_2_/FiO_2 _ratio, disease classification changed in 30% of the patients when changing the FiO_2 _level.

**Conclusion:**

The PaO_2_/FiO_2 _ratio depends on both the FiO_2 _level and the arterial oxygen saturation level. As a minimum, the FiO_2 _level at which the PaO_2_/FiO_2 _ratio is measured should be defined when quantifying the effects of therapeutic interventions or when specifying diagnostic criteria for acute lung injury and acute respiratory distress syndrome. Alternatively, oxygenation problems could be described using parameters describing shunt and ventilation/perfusion mismatch.

## Introduction

The ratio of the partial pressure of oxygen in arterial blood (PaO_2_) to the inspired oxygen fraction (FiO_2_) has been used to quantify the degree of abnormalities in pulmonary gas exchange. The ratio has been used in numerous experimental studies to quantify pulmonary gas exchange before and after therapeutic intervention (for example [1-3]). The PaO_2_/FiO_2 _ratio has also been used in the clinical setting to classify patients' pulmonary gas exchange status, including the definitions of acute lung injury (ALI) (27 kPa ≤ PaO_2_/FiO_2 _< 40 kPa) and of adult respiratory distress syndrome (ARDS) (PaO_2_/FiO_2 _< 27 kPa) [4,5].

Despite its widespread use, the validity of the PaO_2_/FiO_2 _ratio as a tool for assessing pulmonary gas exchange has been questioned. Using mathematical models describing gas exchange, previous authors have simulated values of the PaO_2_/FiO_2 _ratio and have shown them to vary with the FiO_2 _level [6-8]. These theoretical analyses could lead us to believe that the PaO_2_/FiO_2 _ratio is a poor indicator of a patient's pulmonary gas exchange status in the clinic. This hypothesis is only true, however, if the simulations performed are indeed able to describe measured variations in the PaO_2_/FiO_2 _ratio, and if these variations happen within interesting ranges of FiO_2_. The latter of these conditions is crucial in determining whether this ratio is a useful scientific and clinical parameter.

The ability of a particular simulation to accurately describe variation in the PaO_2_/FiO_2 _ratio depends upon the complexity of the mathematical models used. Gowda and Klocke [7] used the complex mathematical model included in the multiple inert gas elimination technique [9] to simulate changes in the PaO_2_/FiO_2 _ratio on varying FiO_2 _levels. This complex model has the advantage of describing pulmonary gas exchange accurately; however, its complexity means that the model is not useful for describing an individual patient in the intensive care unit. Aboab and colleagues used a simple mathematical model where an 'effective' pulmonary shunt was used to describe all ventilation/perfusion (V/Q) abnormalities in the lung [6]. This model has the advantage that values of 'effective shunt' can be estimated from clinical data. Values of 'effective shunt', however, are well known to vary with FiO_2_, as shown previously [10]. A single fixed value of 'effective shunt' may therefore not be able to simulate changes in the PaO_2_/FiO_2 _ratio accurately.

Mathematical models have been proposed recently that describe the gas exchange using two parameters: a shunt value, and a second parameter describing the V/Q ratio [11,12]. These parameter values can be estimated simply and noninvasively in the clinic [13], and have been shown to fit data from a range of mechanically ventilated patients and spontaneously breathing patients [14-16]. These models and techniques therefore provide tools that can both describe pulmonary gas exchange in the individual patient and potentially simulate changes in the PaO_2_/FiO_2 _ratio.

The purpose of the present article is to assess the relevance of variation in the PaO_2_/FiO_2 _ratio with the FiO_2 _level. To do so, we determined whether changes in the PaO_2_/FiO_2 _ratio can be described accurately by either the 'effective shunt' model or a two-parameter model describing shunt and V/Q mismatch. Unlike previous studies that have examined changes in the PaO_2_/FiO_2 _ratio with FiO_2 _theoretically through model simulation [6-8], the present analysis is performed both theoretically and experimentally by comparing model simulations with measured values of the PaO_2_/FiO_2 _ratio in a range of different patients. Simulations of the PaO_2_/FiO_2 _ratio performed with the two-parameter model are compared with those using the 'effective shunt' model to investigate whether the extra complexity of the two-parameter model is justified. The models are then used to simulate whether, and under which conditions, the PaO_2_/FiO_2 _ratio varies with FiO_2_, to further investigate the discrepancies between the two models and whether such variation is clinically relevant.

## Materials and methods

Data were collected from 93 patients, most of these data being published previously [11,14,15]. Patients included postoperative surgical patients following gynaecological laparotomy [11,14] and cardiac surgery [14,15], those patients receiving intensive care therapy [14], normal subjects [14] and patients suffering from cardiac incompensation [14]. Twenty-eight of these patients were mechanically ventilated and presented in the intensive care unit; the remaining 57 patients were breathing spontaneously. Some patients were studied on more than one occasion, giving a total of 120 patient cases. In addition, new data from a further eight mechanically ventilated intensive care patients studied at one or two positive end-expiratory pressure settings were included in the analysis, adding 14 additional patient cases – giving a total of 134 patient cases. All intensive care patients had disorders in pulmonary gas exchange either due to primary infectious involvement or due to a secondary pulmonary involvement as a consequence of severe sepsis or septic shock. Ethical approval was obtained from the relevant ethics committee for all studies, and informed written and oral consent was obtained for all patients.

On each occasion patients were studied using four to eight different FiO_2 _values, achieving arterial oxygen saturation (SaO_2_) values in the range 85–100%. The FiO_2 _values were selected on a patient-specific basis to cover this range, meaning that patients with more severe pulmonary disorders received higher FiO_2 _levels. Steady state was achieved at each FiO_2 _level either by waiting 5 minutes or by the presence of a stable end-tidal oxygen fraction over a 30-second period [13]. At steady-state conditions, measurements were taken of ventilation (FiO_2_, end-tidal oxygen fraction), of end-tidal carbon dioxide fraction, tidal volume, and respiratory frequency, and of arterial acid–base and oxygenation status (SaO_2_, PaO_2_, pH, partial pressure of carbon dioxide, haemoglobin, methaemoglobin, and carboxyhaemoglobin). In some patients it was necessary to administer subatmospheric oxygen fractions to achieve SaO_2 _in the range 85–90%, which was achieved by mixing nitrogen with air in the inspiratory gas. In 18 experiments where all patients were breathing spontaneously, arterial blood gases were only measured at two levels of FiO_2_. These patient cases were excluded from the current analysis, giving a total number of 116 patient cases for data analysis (51 mechanically ventilated patients, 65 spontaneously breathing patients). The PaO_2_/FiO_2 _ratio was calculated at each level of FiO_2_.

### Mathematical models

**Figure 1:**
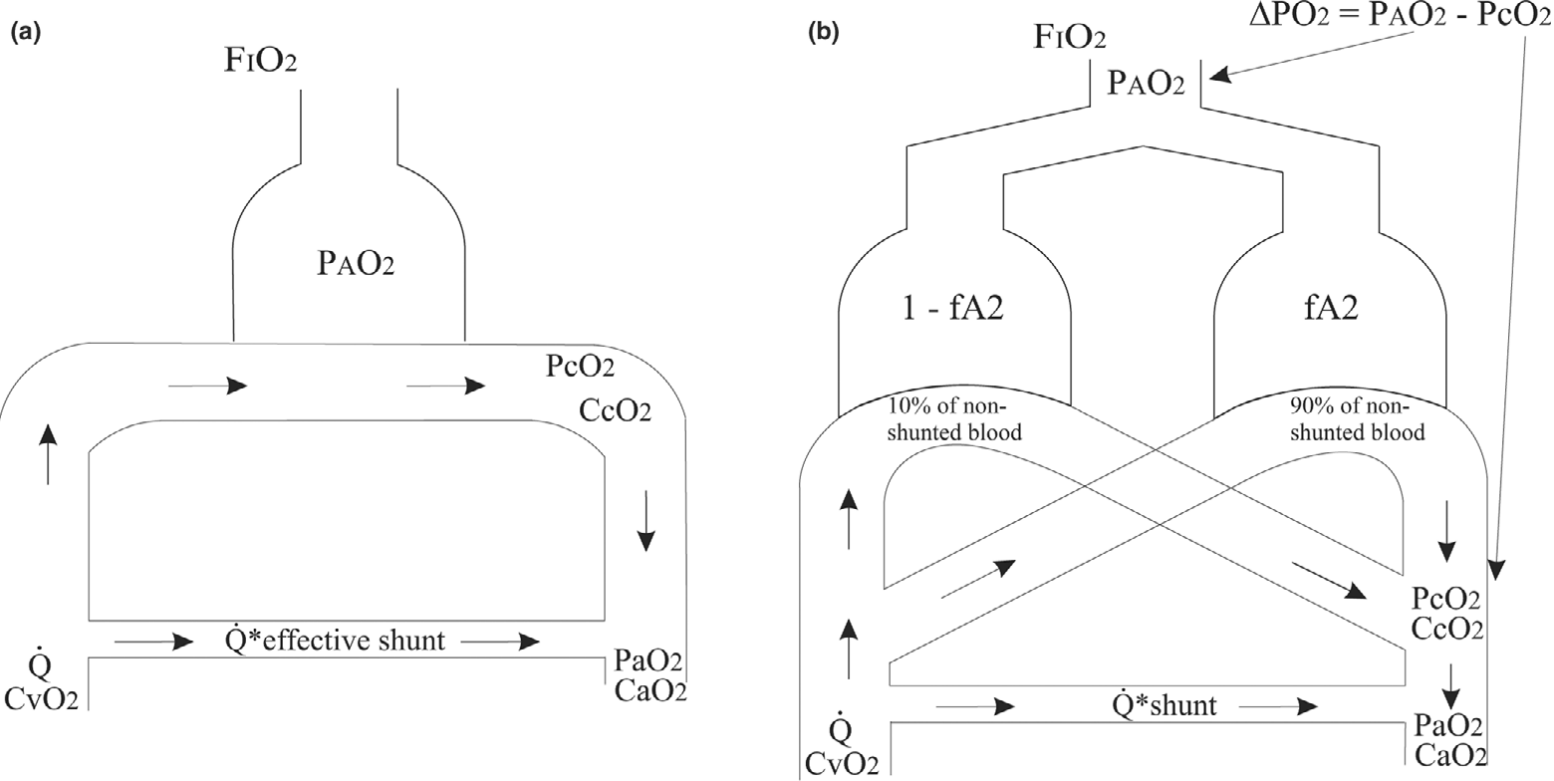
Mathematical models of pulmonary gas exchange. **(a) **The 'effective shunt' model. **(b) **The two-parameter shunt and ventilation/perfusion mismatch model. Data describing oxygen transport in the models are indicated: oxygen partial pressure in alveolar air (P_A_O_2_), oxygen partial pressure in capillary blood (P_c_O_2_), oxygen partial pressure in arterial blood (P_a_O_2_), concentration of oxygen in venous blood (C_v_O_2_), concentration of oxygen in capillary blood (C_c_O_2_), concentration of oxygen in arterial blood (C_a_O_2_), cardiac output (Q), shunt parameter (shunt), and parameters describing ventilation/perfusion mismatch (fA2, ΔPO_2_).

The data were analysed using two mathematical models of gas exchange: the 'effective shunt' model, used by Aboab and colleagues [6]; and the two-parameter model [11,13,14], the equations of which have been published previously ([14] electronic supplement). Figure 1 illustrates how these models differ in their representation of pulmonary gas exchange. The 'effective shunt' model includes one ideally ventilated and perfused alveolar compartment plus a compartment representing pulmonary shunt. The two-parameter model includes two alveolar compartments incorporating V/Q inequality with the addition of a shunt compartment.

In the 'effective shunt' model, oxygenation problems are described by a single parameter ('effective shunt') quantifying the blood flowing through the lungs without being oxygenated. In the two-parameter model, a shunt parameter is included along with the parameter fA2 describing the fraction of ventilation to a compartment receiving 90% of nonshunted perfusion. An fA2 value of 0.9 gives ideal V/Q matching, while lower fA2 values indicate V/Q mismatching. An fA2 value can be transformed into a ΔPO_2 _value, which describes the drop in oxygen pressure from the ventilated alveoli to the mixed blood leaving the lung capillaries; that is, the value in blood prior to the mixing of shunt. As such, ΔPO_2 _describes the extra oxygen pressure required at the mouth to remove an oxygenation problem due to V/Q mismatch; that is, ΔPO_2 _= 20 kPa means air plus 20% inspired oxygen (FiO_2 _= 0.41) is required.

### Mathematical model simulations and statistical analysis

The 'effective shunt' model and the two-parameter model were used in three ways.

A theoretical comparison was performed between model simulations of changes in SaO_2 _and the PaO_2_/FiO_2 _ratio with variation in FiO_2 _using the two mathematical models. To do so, simulations were performed for different values of model parameters.

The models were fitted to the data from each patient in turn using the least-squares method, and the root mean square of the residuals was calculated for each of the fits. Model fits were illustrated by plotting simulated and measured values of SaO_2 _and the PaO_2_/FiO_2 _ratio versus FiO_2_. A statistical comparison of the 'goodness' of fit of the two models to the data was performed using an *F *test [17].

Both models were then used to analyse the variation in the PaO_2_/FiO_2 _ratio over a range of FiO_2 _levels. This analysis had two aims: first, to evaluate the significance of any difference between the two models when fitted to the data; and second, to investigate whether the simulated variation in the PaO_2_/FiO_2 _ratio was relevant. The relevant range was defined on an individual patient basis as the FiO_2 _range that resulted in a simulated value of SaO_2 _within the range 92–98%. The variation in the PaO_2_/FiO_2 _ratio was then used to quantify the number of patients changing disease classification as a result of varying FiO_2 _levels according to the two models across the defined FiO_2 _range, these results being presented in a confusion matrix [18]. Patients were classified into disease groups at the lowest and highest FiO_2 _level in the range, according to the following criteria: ARDS (PaO_2_/FiO_2 _< 27 kPa) [4,5], ALI (27 kPa ≤ PaO_2_/FiO_2 _< 40 kPa) [4,5], and normal (PaO_2_/FiO_2 _> 47 kPa) [19]. Those patients falling outside these categories are defined here as having mild hypoxemia (40 kPa ≤ PaO_2_/FiO_2 _< 47 kPa).

## Results

**Figure 2:**
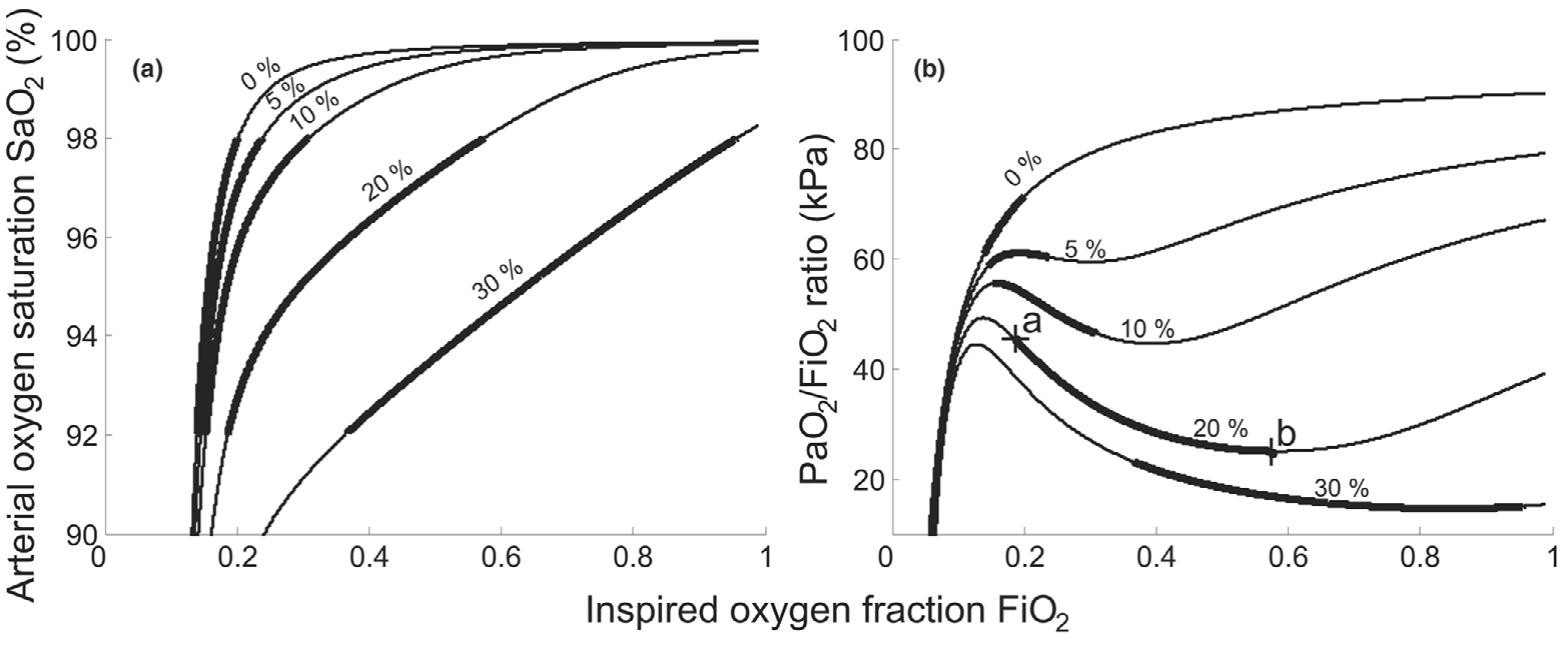
Model simulations of arterial oxygen saturation and arterial oxygen partial pressure/inspired oxygen fraction ratio. **(a) **Inspired oxygen fraction (FiO_2_) versus arterial oxygen saturation (SaO_2_). **(b) **FiO_2 _versus the partial pressure of oxygen in arterial blood (PaO_2_)/FiO_2 _ratio. Simulations performed using shunt = 0–30%, parameter ΔPO_2 _(fA2) = 0 kPa (0.9), oxygen consumption = 0.26 l/min, alveolar minute volume = 5.25 l. Points a and b, the PaO_2_/FiO_2 _ratios for FiO_2 _= 0.19 (point a) and FiO_2 _= 0.57 (point b) – corresponding to the extremes of the relevant range of FiO_2 _(thick solid line).

**Figure 3:**
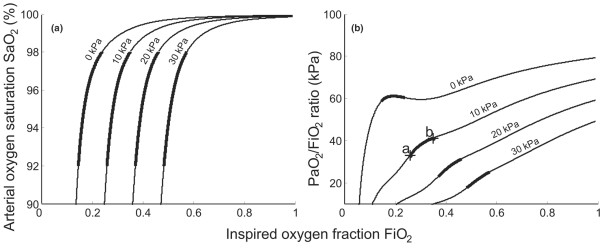
Model simulations of arterial oxygen saturation and arterial oxygen partial pressure/inspired oxygen fraction ratio. **(a) **Inspired oxygen fraction (FiO_2_) versus arterial oxygen saturation (SaO_2_). **(b) **FiO_2 _versus the partial pressure of oxygen in arterial blood (PaO_2_)/FiO_2 _ratio. Simulations performed using shunt = 5%, parameter ΔPO_2 _(fA2) = 0–30 kPa (0.9–0.11), oxygen consumption = 0.26 l/min, alveolar minute volume = 5.25 l. Points a and b, the PaO_2_/FiO_2 _ratios for FiO_2 _= 0.26 (point a) and FiO_2 _= 0.35 (point b) – corresponding to the extremes of the relevant range of FiO_2 _(thick solid line).

Figures 2 and 3 illustrate the results of the theoretical analysis showing the effects of varying FiO_2 _on model simulated values of SaO_2 _and the PaO_2_/FiO_2 _ratio.

Figure 2a,b illustrates the effects of varying either the 'effective shunt' of the model of Aboab and colleagues [6] or the shunt value included in the two-parameter model, these being equivalent for ΔPO_2 _= 0 kPa. Simulated increased shunt depresses the shoulder of the FiO_2 _versus SaO_2 _curve, and depresses and deforms the shape of the FiO_2 _versus PaO_2_/FiO_2 _ratio curve. As a result, the relevant range of FiO_2 _(thick solid part of lines) broadens with increases in shunt. The deformation in the PaO_2_/FiO_2 _ratio curve has a characteristic shape whereby the PaO_2_/FiO_2 _first falls and then gradually rises, explained as follows. On increasing the FiO_2 _level, the partial pressure of oxygen in the lung capillary blood increases. As the lung capillary blood mixes with that shunted, the increase in the partial pressure of oxygen in the lung capillary blood helps to oxygenate the shunted blood, so that the PaO_2 _value increases little and the PaO_2_/FiO_2 _ratio falls. On increasing the FiO_2 _level further, the mixture of shunted and lung capillary blood reaches an SaO_2 _value of about 98% where the arterial blood haemoglobin is almost saturated. Further increases in FiO_2 _translate into increased PaO_2_, and hence an increasing PaO_2_/FiO_2 _ratio. It should be noted that the range of FiO_2 _giving 92–98% saturation may extend below atmospheric oxygen levels (FiO_2 _= 0.21) in patients with only mild gas exchange abnormalities or in normal subjects. The simulations in Figure 2b show how the PaO_2_/FiO_2 _ratio changes with FiO_2 _as found by Aboab and colleagues [6]. For example, for a shunt value of 20% (see Figure 2b, points a and b) the PaO_2_/FiO_2 _ratio falls by 20.5 kPa, from 45.5 kPa to 25 kPa, over the relevant range of FiO_2_.

Figure 3a,b illustrates the effects of varying the degree of V/Q mismatch in the two-parameter model. The effects of a V/Q mismatch on the SaO_2 _or the PaO_2_/FiO_2 _ratio are quite different from the effects of shunt. The FiO_2 _versus SaO_2 _curves are shifted horizontally along the FiO_2 _axis with increasing V/Q mismatch. The PaO_2_/FiO_2 _ratio is increased with increasing FiO_2 _levels, as the absence of significant shunt means that arterial haemoglobin is saturated on small increases in FiO_2_. The small dip in the PaO_2_/FiO_2 _ratio seen in these curves, particularly at the 0 kPa level, is due to the 5% shunt used in these plots. For the cases simulated in Figure 3, the PaO_2_/FiO_2 _ratio is quite sensitive to changes in FiO_2_. Within the relevant range of FiO_2 _(thick solid part of lines) for a ΔPO_2 _value of 10 kPa (see Figure 3b, points a and b), the PaO_2_/FiO_2 _ratio increases by 8.3 kPa, from 32.9 kPa to 41.2 kPa.

**Figure 4:**
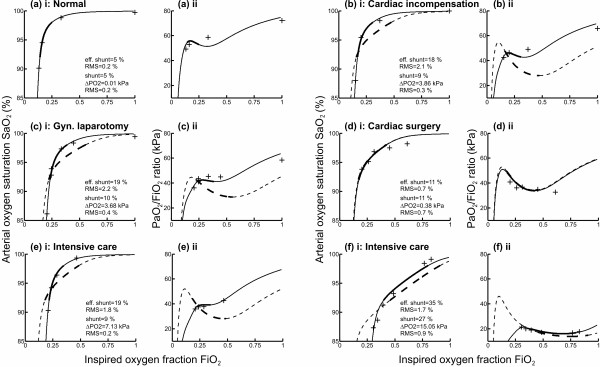
Model simulations and measured data for six patients selected to represent typical cases. Model fitted simulations (curves) and measured data (crosses) describing **(i) **inspired oxygen fraction (FiO_2_) versus arterial oxygen saturation (SaO_2_) and **(ii) **FiO_2 _versus the partial pressure of oxygen in arterial blood (PaO_2_)/FiO_2 _ratio. **(a) **Normal subject [14], **(b) **cardiac incompensation patient [14], **(c) **gynaecological laparotomy patient [14], **(d) **cardiac surgery patient [15], **(e) **intensive care patient [14], and **(f) **previously unpublished intensive care data. Curves, parameter values and fitting residuals (root mean square (RMS)) for the 'effective shunt' model (dashed lines, 'effective shunt' parameter) and for the two-parameter model (solid lines, shunt and ΔPO_2 _parameters). Thick lines, range of FiO_2 _giving a SaO_2 _of 92–98%.

Figure 4 illustrates model simulations and measured data describing the changes in SaO_2 _and the PaO_2_/FiO_2 _ratio on varying the FiO_2 _level for six patients, selected from the 116 patient cases to represent typical cases. Measured values and the model simulations using the 'effective shunt' model and the two-parameter model are shown. The range of FiO_2 _defined for each patient (giving SaO_2 _= 92–98%) is shown, such that those patients with more severe lung diseases have a higher range than those with less severe disease. For each of the fits, values of model parameters are given along with the root mean square value describing the error in model fitting.

The average (± standard deviation) root mean square for fitting the two-parameter model to the data was 0.5 ± 0.4%, compared with 1.4 ± 1.0% (± SD) for the 'effective shunt' model. The results of the *F *test showed that the two-parameter model gave a statistically better fit to the data than the 'effective shunt' model (*P *< 0.005). In all cases the two-parameter model fitted the data either as well as or better than the 'effective shunt' model, as described by the root mean square. In cases where the 'effective shunt' model fitted the data well (for example, Figure 4a,d), the fits of the two models were almost identical. In other cases (for example, Figure 4b,c,e,f), the two-parameter model gave a much better fit to the data.

The plots of FiO_2 _versus the PaO_2_/FiO_2 _ratio illustrated in Figure 4 also show that the two-parameter model is necessary to describe measured changes in the PaO_2_/FiO_2 _ratio, where a V/Q mismatch is present. For some patients (Figure 4b,c,e,f) the measured change in the PaO_2_/FiO_2 _ratio with FiO_2 _had a very different form from that predicted by the 'effective shunt' model. The possibility of a patient being defined in different clinical groups dependent on the FiO_2 _level can be seen, for example, in Figure 4d(ii). Here, according to the two-parameter model, an increase in the FiO_2 _level from 0.21 to 0.43 decreased the PaO_2_/FiO_2 _ratio from 45 kPa to 34 kPa, resulting in a change in disease classification from mild hypoxemia to ALI.

Table 1 presents a confusion matrix showing the number of patient cases classified in the four disease groups and how this classification varied with changes in FiO_2 _using the two models. The left-hand column presents the number of patient cases classified in each group at a low FiO_2 _level. The table elements then describe the patient cases classified in each group at high FiO_2_. Each element can therefore be interpreted to illustrate movement between groups; for example, of the 56 patient cases classified as normal at low FiO_2 _level using the two-parameter model, 39 patients remain classified as normal at high FiO_2 _levels.

**Table 1:** Numbers of patients changing disease group with increasing inspired oxygen fraction (FiO_2_) across the relevant range

Low FiO_2_	High FiO_2_			
	
	Normal	Mild hypoxemia	Acute lung injury	Acute respiratory distress syndrome
'Effective shunt' model	*n *= 23	*n *= 15	*n *= 40	*n *= 38
Normal (*n *= 64)	23	14	27	0
Mild hypoxemia (*n *= 20)	0	1	13	6
Acute lung injury (*n *= 14)	0	0	0	14
Acute respiratory distress syndrome (*n *= 18)	0	0	0	18
Ventilation/perfusion and shunt model	*n *= 42	*n *= 19	*n *= 31	*n *= 24
Normal (*n *= 56)	39	12	5	0
Mild hypoxemia (*n *= 19)	3	6	9	1
Acute lung injury (*n *= 23)	0	1	16	6
Acute respiratory distress syndrome (*n *= 18)	0	0	1	17

Patients classified into disease groups at the lowest and highest FiO_2 _level in the range, according to the following partial pressure of oxygen in arterial blood (PaO_2_)/FiO_2 _ratio criteria: normal (PaO_2_/FiO_2 _> 47 kPa) [19], mild hypoxemia (40 kPa ≤ PaO_2_/FiO_2 _< 47 kPa), acute lung injury (27 kPa ≤ PaO_2_/FiO_2 _< 40 kPa) [4,5], and acute respiratory distress syndrome (PaO_2_/FiO_2 _< 27 kPa) [4,5].

Disease classification changed in 60 of 116 patient cases (~50%) according to the 'effective shunt' model, compared with 38 of 116 patient cases (~30%) according to the two-parameter model. With an increase in the FiO_2 _level, but maintaining SaO_2 _within the range 92–98%, according to the 'effective shunt' model the number of patient cases classified as ALI and ARDS changed from 14 to 40 (~186% increase) and from 18 to 38 (~111% increase), respectively. According to the two-parameter model, the number of patient cases classified as ALI and ARDS changed from 23 to 31 (~35% increase) and from 18 to 24 (~33% increase), respectively. According to the 'effective shunt' model, disease severity only increased with FiO_2 _– whereas five patient cases changed classification to a less severe disease group according to the two-parameter model.

## Discussion

The present study has investigated the variation in the PaO_2_/FiO_2 _ratio with FiO_2_, and the mathematical model complexity necessary to describe this variation. For the first time this analysis has been performed not only theoretically using mathematical model simulations, but also experimentally from measurements of the PaO_2_/FiO_2 _ratio taken at different FiO_2 _levels.

The use of a two-parameter model of gas exchange to describe variation in the PaO_2_/FiO_2 _ratio has been investigated. This model has been shown, using an *F *test, to provide a statistically better fit to oxygenation data than an 'effective shunt' model, even when taking into account the degrees of freedom lost due to the presence of an extra parameter. This improvement in fit can be seen in the plots shown in Figure 4, which were selected to illustrate a variety of patient cases. In four of these six cases (Figure 4b,c,e,f), simulations using the 'effective shunt' model do not describe the measured variation in the PaO_2_/FiO_2 _ratio with varying FiO_2 _level. Interpretation of the PaO_2_/FiO_2 _ratio changes in these four examples using the 'effective shunt' model would result in an overestimation of the changes in the PaO_2_/FiO_2 _ratio when varying the FiO_2 _level. In the remaining two cases (Figure 4a,d) the 'effective shunt' model and the two-parameter model provide an equivalent description of the data. The case presented in Figure 4a represents a normal subject with no V/Q mismatch problem and 5% shunt, whilst the case shown in Figure 4d represents a patient with little V/Q mismatch such that all oxygenation problems can be explained by shunt.

In general, use of the 'effective shunt' model to simulate changes in the PaO_2_/FiO_2 _ratio results in an overestimate of the number of patient cases changing disease classification upon increasing FiO_2_, as illustrated in Table 1. Approximately 50% of the patient cases change classification using the 'effective shunt' model, in comparison with 30% using the two-parameter model.

For five patient cases, the change in disease classification simulated by the 'effective shunt' model was in the opposite direction to that shown by the measured PaO_2_/FiO_2 _ratio – the 'effective shunt' model simulating an incorrect degree of disease severity. In these patient cases the V/Q mismatch was the major cause of hypoxemia according to the two-parameter model, and this model was necessary to simulate these changes in the PaO_2_/FiO_2 _ratio. The difference in the direction of disease classification provided by these two models can be understood by looking at Figure 4e. For this case, the 'effective shunt' model simulation would result in a reduction in the PaO_2_/FiO_2 _ratio on increasing the FiO_2 _level in comparison with both the raw data and the two-parameter model simulation.

The necessary criteria for diagnosing ALI and ARDS include acute onset of respiratory failure, bilateral infiltrates seen on a frontal chest radiograph and no clinical evidence of left atrial hypertension in addition to the PaO_2_/FiO_2 _ratio limits [4]. In the present study, patient cases were classified only from the PaO_2_/FiO_2 _ratio. The sole difference between the criteria for ALI and ARDS, however, is the level of hypoxemia quantified by the PaO_2_/FiO_2 _ratio.

## Conclusion

The present article has shown that the PaO_2_/FiO_2 _ratio depends on both the FiO_2 _level and the SaO_2 _level, and that, for changes in FiO_2 _corresponding to an SaO_2 _range of 92–98%, 30% of patients change disease classification due to variation in the PaO_2_/FiO_2 _ratio. The clinical and scientific utility of the PaO_2_/FiO_2 _ratio therefore seems doubtful, and at the very least the FiO_2 _level at which the PaO_2_/FiO_2 _ratio is measured should be specified when quantifying the effects of therapeutic interventions or when specifying diagnostic criteria for ALI and ARDS. Perhaps more appropriate would be to replace the single-parameter PaO_2_/FiO_2 _ratio description with two parameters, a parameter to describe the oxygenation problem due to V/Q mismatch and one to describe oxygenation problems due to shunt. Indeed, Riley and Cournand [20] recognized in the 1950s that oxygenation problems should be divided in this way. With the ability to identify two-parameter models rapidly using pulse oximetry data [14] and simple clinical methods [13], their clinical application seems timely.

## Key messages

• The variation in the PaO_2_/FiO_2 _ratio with the FiO_2 _level is scientifically and clinically relevant.

• The variation in the PaO_2_/FiO_2 _ratio with the FiO_2 _level cannot be explained with an 'effective shunt' model, and requires a more complex, two-parameter, model.

## Abbreviations

ALI = acute lung injury; ARDS = acute respiratory distress syndrome; ΔPO_2 _= drop in oxygen pressure from the ventilated alveoli to the mixed blood leaving the lung capillaries oxygen; fA2 = fraction of ventilation to a compartment receiving 90% of nonshunted perfusion; FiO_2 _= inspired oxygen fraction; PaO_2 _= partial pressure of oxygen in arterial blood; SaO_2 _= arterial oxygen saturation; V/Q = ventilation/perfusion.

## Competing interests

SK, SA and SER are all shareholders of Mermaid Care APS, a company involved in the development of equipment for the measurement of pulmonary gas exchange. SA is a board member of Mermaid Care APS. All other authors declare that they have no competing interests.

## Authors' contributions

All authors contributed to the conception and design of the study. SK, KE and CA contributed to the data collection and clinical interpretation of the results. DSK, BWS, SA and SER contributed to the mathematical modelling, data analysis and technical interpretation of the results, including statistical analysis. DSK and SER drafted the manuscript, with all other authors being involved in its revision and approval.
